# IGSA: Individual Gene Sets Analysis, including Enrichment and Clustering

**DOI:** 10.1371/journal.pone.0164542

**Published:** 2016-10-20

**Authors:** Lingxiang Wu, Xiujie Chen, Denan Zhang, Wubing Zhang, Lei Liu, Hongzhe Ma, Jingbo Yang, Hongbo Xie, Bo Liu, Qing Jin

**Affiliations:** College of Bioinformatics Science and Technology, Harbin Medical University, Harbin, Heilongjiang, China; Universita degli Studi di Torino, ITALY

## Abstract

Analysis of gene sets has been widely applied in various high-throughput biological studies. One weakness in the traditional methods is that they neglect the heterogeneity of genes expressions in samples which may lead to the omission of some specific and important gene sets. It is also difficult for them to reflect the severities of disease and provide expression profiles of gene sets for individuals. We developed an application software called IGSA that leverages a powerful analytical capacity in gene sets enrichment and samples clustering. IGSA calculates gene sets expression scores for each sample and takes an accumulating clustering strategy to let the samples gather into the set according to the progress of disease from mild to severe. We focus on gastric, pancreatic and ovarian cancer data sets for the performance of IGSA. We also compared the results of IGSA in KEGG pathways enrichment with David, GSEA, SPIA, ssGSEA and analyzed the results of IGSA clustering and different similarity measurement methods. Notably, IGSA is proved to be more sensitive and specific in finding significant pathways, and can indicate related changes in pathways with the severity of disease. In addition, IGSA provides with significant gene sets profile for each sample.

## Introduction

During the past several years, the measurement of differential gene expression has been applied to various high-throughput biological studies. To facilitate the functional analysis of long lists of genes [1], a number of enrichment methods, including Over-Representation Analysis (ORA), Functional Class (FCS) approaches, Network-Based (NB) approaches[2], Single Sample (SS) methods[3] and others (such as topGO [4]) have been developed. ORA, the earliest approach [5], statistically evaluates the fraction of genes in gene sets (e.g., pathways) found among the set of significant differentially expressed genes. The methods in this category include DAVID [6], Onto-Express [7], and GoMiner [8]. The hypothesis of FCS approaches is that changes in sets of functionally related genes can have significant effects; this approach helps to alleviate the need for selecting significant genes as a first step, which omits important information about the genes. FCS methods include GSEA [9] and PAGE [10]. NB approaches introduce networks (e.g., pathway network, gene interaction network, etc.) to promote enrichment analysis. A variety of tools are based on the NB approach, such as SPIA (pathway topology based) [11], DRAGEN (transcriptional regulatory network based) [12], ScorePAGE (pathway topology based) [13], and ToppCluster [14]. Single Sample (SS) methods compute gene set scores for each individual sample based on the expression of genes; such methods include PLAGE [15], ssGSEA [16], Z-score [17], and GSVA [18].

However, one weakness in these methods is that many of them neglect information about the heterogeneity of gene expression in the samples [19], which may lead to the omission of some specific gene sets. Moreover, clustering approaches based on traditional methods, such as unsupervised hierarchical clustering, may classify samples (or gene sets) meaningfully, although these methods hardly reflect the different severities of diseases in different classes. Hence, these tools are inappropriate for analysis in some situations, particularly in individualized medicine.

Here, we present Individual Gene Sets Analysis (IGSA), a functional and efficient software tool based on Java and R, which can be used to analyze gene lists and visualize the results from various angles. IGSA takes advantage of the heterogeneity of gene expressions in samples and the homogeneity of gene expression at the functional level.

Differing from other methods such as ORA, FCS and NB, whose enrichment analysis is based on the changes of gene expression between cases and controls, IGSA preserves all gene expression data in samples and involves these data in the calculation of an expression score for each gene set in each sample. This approach is similar in spirit to SS; however, methods in SS, such as PLAGE, standardize gene expression profiles (GEPs) through z-scores, which assumes that gene expression profiles are jointly normally distributed; this means that all genes act independently within each gene set. Moreover, methods such as ssGSEA and GSVA rank gene expression and expression-level statistics, respectively, resulting in the loss of detailed information about gene expression in each sample.

**Fig 1:**
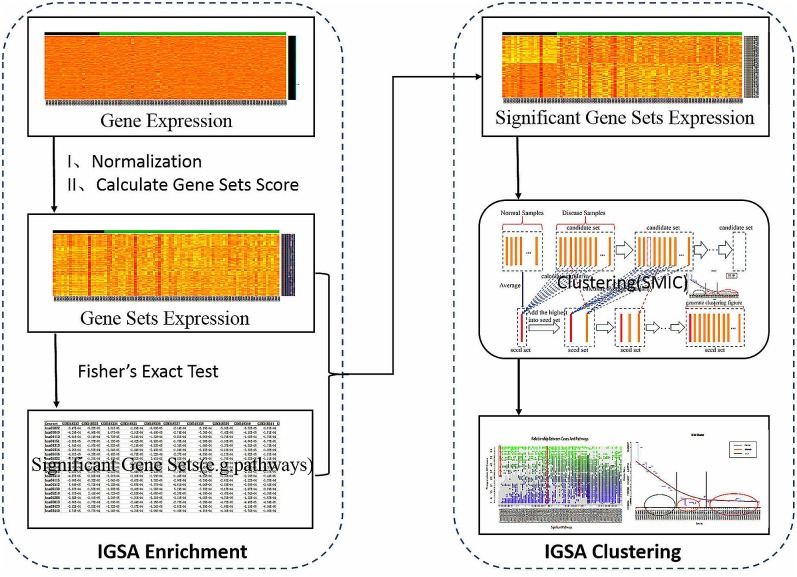
The workflow of IGSA. Step one, normalize the expression of genes and calculate each gene set expression score for each sample. Step two, find significant gene sets (e.g., pathways) according to Fisher’s exact test (count the number of gene set scores above or below the average score in controls and cases, and construct 2x2 contingency tables). Step three, obtain significant gene set expression according to a list of significant gene sets and gene sets expression, and then subject the results to IGSA clustering (the similarity measure is SMIC).

In addition, IGSA employs the accumulating clustering strategy (Fig 1), letting samples (cases) gather into the set according to the degree of possible deterioration, which may reflect the severity of a disease in different samples. Furthermore, the similarity measurement used in IGSA clustering focuses more on the similarity between the samples in quality (i.e., coordinated expression change (up-regulated or down-regulated) in gene sets between two samples) rather than in quantity (i.e., the similarity of gene set expression values between two samples), which promotes the effect of clustering compared with other methods (e.g., Pearson's correlation, etc.).

We used IGSA to analyze several cancer-related datasets (e.g., gastric cancer, pancreatic cancer and ovarian cancer). Compared with other methods (DAVID, GSEA, SPIA, GSVA, ssGSEA and Z-score), besides the gene sets related to many cancers, this method identified many specific gene sets in each cancer and, to a certain extent, indicated changes in gene sets related to the severity of different cancer. IGSA can be accessed freely at http://210.46.85.180:8080/IGSAWeb.

## Materials and Methods

### Data preparation

#### Gastric cancer data

The gastric cancer data (GSE13861) were obtained from GEO. There were a total of 84 samples, and among these, 19 were classified as normal samples.

#### Pancreatic cancer data

The pancreatic cancer data (GSE16515) were obtained from GEO. There were a total of 52 samples, with 16 samples consisting of both tumor and normal expression data and 20 samples consisting of only tumor data.

#### Ovarian cancer data

The ovarian cancer data (TCGA_OV_exp_u133a) were accessed from TCGA. The batch 9 ovarian cancer dataset consists of 46 samples, including 8 normal samples and 37 ovarian cancer samples (the remaining one sample was discarded because it is not normal or cancer). The data were downloaded in September 2014. The batch 40 ovarian cancer dataset included a total of 51 ovarian cancer samples. The survival analysis was based on samples that were diagnosed after the year 2000.

#### Hepatitis data

These data (GSE48452) were available from GEO, including 73 samples of human liver grouped into C (control = 14), H (healthy obese = 27), S (steatosis = 14) and N (nash = 18) groups.

#### Pathways

There were a total of 278 pathways (version 70.0) obtained from KEGG.

#### GO gene sets

We downloaded the gene ontology data (data-version: releases/2015-08-20) and the annotation data (gaf-version: 2.0) from the Gene Ontology Consortium. The third-layer GO terms of BP (Biological Process) and MF (Molecular Function) were extracted and annotated with gene product annotation. This analysis included 1,334 BP terms and 331 MF terms.

### IGSA enrichment

The following procedure was adopted for IGSA enrichment.

#### Normalize the gene expression

All of expression data were transformed to the log scale and without any filtering for the very low expressed genes. We removed the probes which could not be mapped to any genes or mapped to multiple genes.

Taking into account differences in gene expression among the samples (disease samples and normal samples), we calculated the proportions of gene expression in each sample and then compared the proportions with the average level.

Let g_ij denote the expression value of gene i in sample j. Let N be the number of samples. Then, the normalized expression value of each gene in each sample (Expr_Gene(g_ij)) is calculated as follows:

$${\text{Expr}}_{\text{Gene}\left({\text{g}}_{\text{ij}}\right)}=\frac{{\text{g}}_{\text{ij}}}{\sum_{\text{j}}^{\text{N}}{\text{g}}_{\text{ij}}}-\frac{1}{\text{N}}$$

In this way, the expression values of genes higher than the average level are intended to be positive (also called up-regulated), and the values of genes lower than the average level are inclined to be negative (down-regulated).

#### Calculate gene set expression in different samples

We used the average expression values of genes in both the gene set and the sample (i.e., the intersection of genes from the sample and from the gene set) as the gene set (e.g., pathway) expression in the sample.

Let Q_i denote all genes in the gene set i (e.g., pathway), and let S_j be the all genes in sample j. Let | Q_i ∩ S_j | denote the number of genes which exist in both the sample j and the gene set i. The expression of gene set i (e.g., pathwayP) of sample j〖Expr_GeneSet〗_pathway(P_(i, j)) can be calculated by gene expression (denote as Expr_Gene) using the following formula:

$$Expr_{GeneSet}{}_{pathway}\left(P_{i,j}\right)=\frac{\sum Expr_{Gene\left(Q_{i}\cap S_{j}\right)}}{\left|Q_{i}\cap S_{j}\right|}$$

#### Calculate the significance of each gene set

For each gene set, we counted the number of expression scores above or below the average value (the average value was zero after normalization applied with method 2.2.1) in controls and cases. Then, we constructed 2x2 contingence tables and used Fisher’s exact test to obtain the significance value for each gene set. The null hypothesis is that the gene set (i.e., pathway) is independent of the cancer. We also adjusted the p value of each gene set by the FDR.

### IGSA clustering

In consideration of the amplification of gene expression along with the progression of cancer (more genes tend to be expressed aberrantly) [20], we developed an accumulating clustering strategy that can reflect the degree of disease severity by adding disease samples into the seed set according to the average similarity of the disease sample with other seeds (samples as start seed and the other seeds in the seed set).

#### Similarity measurement in IGSA clustering (SMIC)

The similarity measurement in IGSA clustering focuses more on the similarity between the samples in quality (i.e., coordinated changes such as up-regulated or down-regulated expression in gene sets between two samples) rather than in quantity. We used qualitative rather than quantitative measures to avoid the impact on the analysis that results from the over-fitting model.

The similarity between two samples in IGSA clustering is calculated based on the co-expression of gene sets (CGSs, gene sets in both samples whose expression values are simultaneously above or below the average value) and differential expression of gene sets (DGSs, gene sets in both samples whose expression values are on two sides of the average values).

The similarity between two samples can be simply represented as the number of CGSs. However, the expression values of some CGSs in both samples may be simultaneously above or below the average value; this indicates that the phenomenon of co-expression among some gene sets may be a random event, which is common when the number of samples is not large enough; this phenomenon is also observed for DGSs. As a result, a weight (the probability of a gene set whose expression in both samples is not a random event, in which 1-means the probability of a gene set whose expression in both samples is a random event) should be introduced into the calculating to relieve the impact of the pseudo CGSs and DGSs.

Let N_cgs denote the number of CGSs and N_dgs denote the number of DGSs. Thus, the number of real CGSs in CGSs is N_cgs*θ^(N_cgs) (Only the gene set in CGSs whose expression is not a random event will be regarded as a real CGS), and the number of real CGSs in DGSs is N_dgs*〖(1‒θ)〗^(N_dgs) (For a gene set in DGSs whose expression is random, this may indicate co-expression). The complete function of similarity measurement (〖Similarity〗_ij) for sample i and sample j is as follows:

$${\text{Similarity}}_{\text{ij}}={\text{N}}_{\text{cgs}}{*\theta }^{{\text{N}}_{\text{cgs}}}+{\text{N}}_{\text{dgs}}*{\left(1-\theta \right)}^{{\text{N}}_{\text{dgs}}}$$

The interval of θ is [0.5, 1] (We assumed that most co-expression events should be real, and thus the value of theta should not be less than 0.5). When θ is 0.5, all gene sets are expressed at random, and when θ equals 1, all CGSs and DGSs are considered to be meaningful.

Furthermore, due to the uncertainty of θ, which may be interfered by array signal quality, etc., we use the maximum likelihood estimation (MLE) method to calculate the similarity.

$${\text{Similarity}}_{\text{ij}}=\text{L}\left(\theta \right)={\text{N}}_{\text{cgs}}{*\theta }^{}+{\text{N}}_{\text{dgs}}*{\left(1-\theta \right)}^{}$$

#### The concrete steps in IGSA clustering

**I**GSA clustering is based on the similarity of expression of significant gene sets derived from IGSA enrichment.

**Step 1:** Create a seed set for storing seed samples that represent the start seed and samples that have been clustered and create a candidate set for storing candidate samples (disease samples) that are waiting to be clustered.

**Step 2:** Construct a vector as a start seed through calculating the average expression value of each gene set (i.e., significant differential expression pathways) in normal samples, and add the vector into the seed set (the vector is a start seed). Add all disease samples into the candidate set. For example, suppose after IGSA enrichment, we obtain 11 significant gene sets. We first obtained the start seed through calculating the average expression value of these 11 gene sets in all normal samples and added it into the seed set as the start seed.

**Step 3:** Calculate the average similarity score of each sample in the candidate set with all seeds, and then add the sample, rather than the gene-set, with the highest average similarity score into the seed set (the sample is marked as seed) and remove the sample from the candidate set. For example, suppose we have 20 cancer samples in the candidate set and 2 samples in the seed set (the one is the start seed and the other is the clustered disease sample of the highest similarity score with the start seed). For each of the 20 cancer samples, we calculate the average similarity between the cancer sample and 2 samples in the seed set. Finally, we will obtain 20 similarity values for 20 cancer samples and we will move the sample with the highest value into the seed set from the candidate set. Thus, the size of the seed set is increased to 3, and the size of candidate set is decreased to 19.

**Step 4:** Repeat step 3 until the candidate set is null.

**Fig 2:**
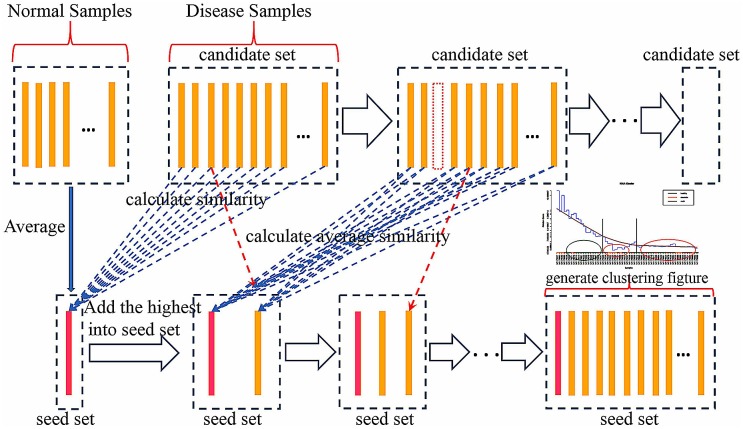
The workflow of IGSA clustering. Step one, create an empty seed set and an empty candidate set. Step two, construct a start seed by calculating the average expression value of each significant gene set in the normal samples, and add the start seed into the empty seed set. Add all of the disease samples into the empty candidate set. Step three, calculate the average similarity of each sample in the candidate set with all seed samples in the seed set, and move the sample with the highest similarity score from the candidate set to the seed set. Step four, repeat step three until the candidate set is null.

All samples are clustered in turn according to the similarity score. Fig 2 shows the workflow of IGSA clustering; this can be used for gene set clustering as well. The procedure is similar to sample clustering except the calculation of similarity is based on gene set expression in each sample rather than based on sample expression in each gene set, and the start seed is a zero-vector (there is no tag in the gene sets that marks each of them as normal or diseased as in the sample; therefore, the start seed is set as a zero-vector, and the length of the vector is equal to the number of samples).

## Results

### IGSA enrichment results

To evaluate the performance of IGSA enrichment, we ran the enrichment analysis program based on the pathways involved in various cancer-related expression profiles.

We first applied this method to a gastric cancer dataset, and IGSA (FDR<0.01) identified 48 significantly up-regulated pathways (SUPs; pathways whose expression value was higher than the average expression value) and 38 significantly down-regulated pathways (SDPs, the pathways whose expression value was lower than the average expression value). Out of 48 SUPs found by IGSA, 35 pathways were reported to be related to gastric cancer (the accuracy was approximately 72.92%; we denote accuracy as the proportion of the found pathways reported to be related to the cancer by manually finding supporting papers with research based on experiments). The accuracy of SDPs identification was approximately 76.32%.

In addition to the common cancer-related pathways (such as cell cycle, DNA replication, etc.), IGSA also found some pathways that were specifically related to gastric cancer. For example, the primary pathway for bile acid biosynthesis (hsa00120), in which bile acid promotes gastric carcinogenesis without inflammatory cell infiltration [21]; the other specific pathways, like Notch signaling pathway (hsa04330) [22], salmonella infection (hsa05132) [23], gastric acid secretion (hsa04971) [24], were also identified using IGSA.

Our methods were also applied to pancreatic cancer and ovarian cancer datasets to demonstrate the universality of IGSA. Significantly, at an FDR<0.01, IGSA found 37 SUPs (the accuracy was 78.38%) and 6 SDPs (the accuracy was 83.33%) in the pancreatic cancer dataset and 18 SUPs (the accuracy was 72.22%) and 28 SDPs (the accuracy was 53.57%) in the ovarian cancer dataset. S1–S3 Tables shows all significant differentially expressed pathways found in the three cancer-related datasets and related supporting papers.

### Comparisons with other methods

**Fig 3:**
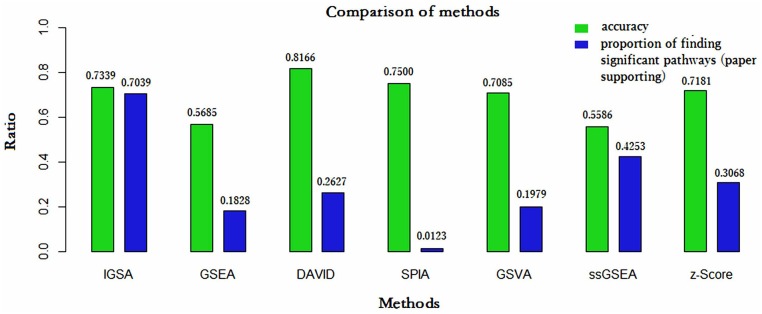
The comparison of the seven methods by accuracy (average accuracy in three cancer-related datasets) and the proportion of significant pathways supported by papers found in three cancer-related datasets. IGSA was more robust and sensitive in finding significant pathways compared with the other methods. Although the accuracy of DAVID and SPIA was a bit higher than that of IGSA, both DAVID and SPIA found only a subset of significant pathways.

We compared the IGSA method with other enrichment methods, including DAVID, GSEA, SPIA, GSVA, ssGSEA and Z-score (Fig 3).

Most of these methods identified some pathways commonly related to cancers, including cell cycle (hsa04110) and DNA replication (hsa03030). However, IGSA also found pathways that were specifically related to the different cancer types.

Three methods (IGSA, DAVID and GSEA) performed well in gastric cancer data (accuracy > 70%) (S4 Table online), with the exception of GSEA in finding SDPs (no significant pathways found). Notably, more than 95% of papers supporting significant pathways were found by IGSA (S5 Table).

In pancreatic cancer datasets, most of the tested methods identified some significant pathways, except for SPIA (found no SUPs and only one SDP), ssGSEA (the accuracy was less than 70%) and GSVA (found no SDPs). Again, IGSA identified the most paper-supported significant pathways (the proportion was approximately 73.91%).

The methods performed less well in finding pathways from the ovarian cancer dataset (TCGA, Batch9); however, compared with the other methods, DAVID and IGSA still performed well in identifying SUPs (accuracy > 70%). Although DAVID was 100% accurate in finding SDPs, this method found only 5 pathways, which was fewer than IGSA (16 pathways).

S1–S3 Tables show the list of significant pathways (including related supporting papers) found by the seven methods. S4 and S5 Tables show the comparison of the seven methods by accuracy and the proportion of significant pathways supported by papers that each method identified.

### Potential progression of diseases at a functional level

**Fig 4:**
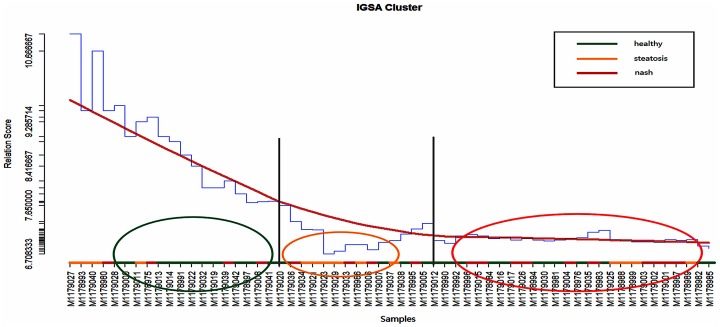
The clustering of samples in hepatitis datasets. The blue curves show the average similarity scores of clustering samples. The red curves were loess curves obtained by fitting the similarity scores. The gray vertical lines are used to divide the samples according to the flex points in the red curve. Most serious disease samples (nash) tended to be clustered on the right (in class 3). In class 2, Samples of less severe disease (steatosis) showed a tendency to cluster in the middle. In class 1, most of healthy obese samples tended to be clustered on the left. To some extent, the clustering may reveal the severity of samples in hepatitis datasets.

We applied the IGSA clustering to a hepatitis dataset (P<0.05), and 14 significant pathways were found (Fig 4). As expected, IGSA clustering was capable of indicating the progression of hepatitis. In Fig 4, samples with the most severe disease clustered at the end (colored red). The other two types of sample, although not clustered well (perhaps caused by pseudo-significant pathways), were still distinguished by the progression of disease to some extent.

**Fig 5:**
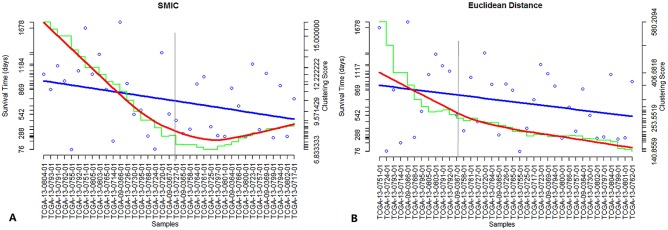
The comparison of IGSA clustering with different similarity measurement. The blue points represent the survival time of the samples. The blue lines were generated by linear fitting the blue points. The green curve shows the average similarity scores of clustering samples. The red curves were loess curves obtained by fitting the similarity scores. The gray vertical lines are used to distinguish the samples according to the flex points in the red curve. (A) represents the IGSA clustering based on SMIC applied in the ovarian cancer data set (batch 9) based on pathways. (B) represents the IGSA clustering based on Euclidean distance applied in the ovarian cancer data set (batch 9) based on pathways. The survival time in both methods (A, B) tended to decrease. However, the red curve in B was too smooth to divide the samples into different disease classes.

We also applied IGSA clustering to the ovarian cancer dataset (TCGA, batch9). In clustering based on SUPs, the survival time was reduced when newer cancer samples were added into the seed set. In clustering based on all significant pathways (including SUPs and SDPs), the clustering was not significant. One possible reason for this result is the high false-positive rate among SDPs (the accuracy was only approximately 53.57%). We also compared the similarity measurement in IGSA clustering (SMIC) with other methods, including Euclidean distance, Pearson's correlation and Spearman's rank correlation (Fig 5, Figure A in S1 File). The results showed that the IGSA clustering based on three measurement (SMIC, Pearson's correlation and Spearman's rank correlation) all can cluster the disease samples into different classes, except that the clustering based on Euclidean distance didn’t perform well (p value of 0.061). Further studies on these classes (obtained by the IGSA clustering based on SMIC, Pearson's correlation and Spearman's rank correlation) by performing survival analysis indicated that the SMIC (p value of 0.018), compared with Pearson's correlation (p value of 0.049) and Spearman's rank correlation (p value of 0.049), was more remarkable, although the clustering based on Pearson's correlation and Spearman's rank correlation can cluster the disease samples significantly too.

**Fig 6:**
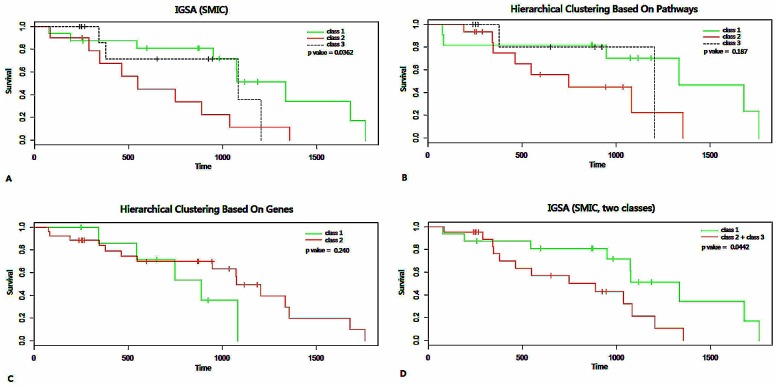
The classification comparison of IGSA, HCBP (hierarchical clustering based on pathways) and HCBG (hierarchical clustering based on genes) in ovarian cancer datasets (TCGA batch 9). A shows the survival time curves of three classes obtained by IGSA (p value of 0.0362). B shows the survival analysis of three classes obtained by HCBP (p value of 0.187). C shows the survival time curves of three classes obtained by HCBG (p value of only 0.240). D shows the survival time curves of two classes (class 1 and class 2,3) obtained by IGSA (p value of 0.0362). The p values in both A and D are significant compared with HCBP and HCBG.

To analyze the significant pathways found by IGSA enrichment, we first used unsupervised hierarchical clustering (UHC) to cluster the samples (from ovarian cancer dataset, TCGA, batch9) based on the pathways. We evaluated the clustering of the ovarian cancer data through the corresponding survival analysis. In consideration of the low accuracy in finding SDPs (only 53.57%), we applied the clustering to the samples based on SUPs and divided the cases into three classes according to their threshold for distinguishing controls from cases. We also compared the result with clustering based on the up-regulated genes (SAM, FDR<0.1, clustered by unsupervised hierarchical clustering, and the samples were divided into two classes according to their threshold for distinguishing controls from cases). The result based on pathways (p value of 0.187) was marginally better than the other (p value of 0.240) (Fig 6B and 6C, Figure B in S1 File).

Further improvement was made when we divided the cases into three classes based on the results of IGSA clustering (the classes were divided according to the flex points in the loess curve fitted to the adjacent similarity scores of clustering samples where the changes of similarity in samples from both sides were distinctly distinguished; Fig 6A). Interestingly, the three classes were rather similar to the three classes obtained by pathway-based UHC (the average ratio of overlap in three classes was approximately 32.21%). However, compared with the results of survival analysis using UHC, the results of IGSA were more significant (p value of 0.0362). In addition, according to the results of the survival analysis, we found that the curve of class 3 was not distinctly separated from the other two classes (although the probability for surviving more than 1,300 days in class 3 was lower than in the other two classes). Therefore, we integrated the samples from class 2 and class 3 into one class and conducted the survival analysis again. Notably, the result was still significant (p value of 0.0442), and the survival time from the two classes was distinct (Fig 6D).

**Fig 7:**
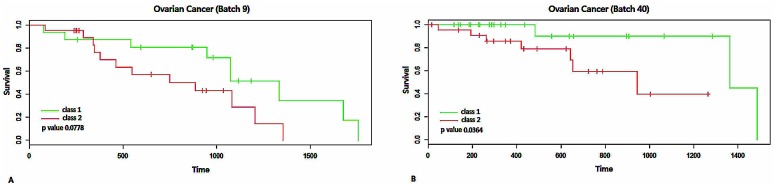
The survival analysis of ovarian cancer datasets (TCGA batch 9 and batch 40). Part A shows the survival time curves of two classes obtained by IGSA (p value of 0.0778). Part B shows the survival analysis of two classes obtained by IGSA based on the same significant pathways (13 paper supporting SUPs and paper supporting 16 SDPs, p value of 0.0364).

Furthermore, we used IGSA clustering to cluster the samples based on the expression profiles of significant pathways (including 13 SUPs and 16 SDPs found by IGSA), which were shown to be related to ovarian cancer in previously published papers. The samples were divided into two classes, and the survival time in the two classes was significantly different (p value of 0.0778. Fig 7A, Figure C(A) in S1 File). To evaluate the applicability of the significant pathways (i.e., whether these significant pathways could be used as a signature for ovarian cancer), another ovarian cancer dataset (TCGA, ovarian cancer data, batch 40) was subjected to clustering based on the same significant pathways. Interestingly, the samples in the data were also divided into two classes, and according to the survival analysis, the difference between the two classes was significant (p value of 0.0364. Fig 7B, Figure C(B) in S1 File).

**Fig 8:**
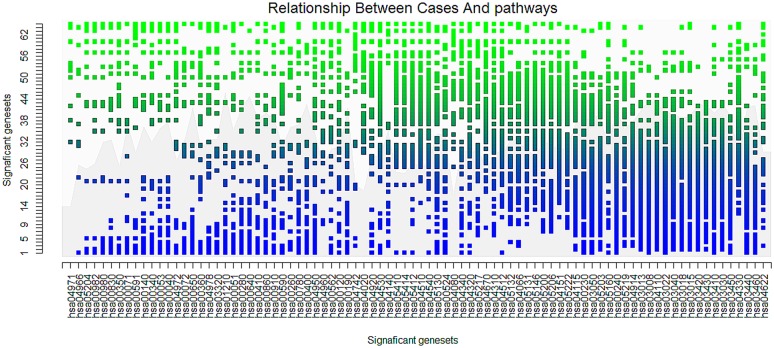
The clustering of samples and significant pathways in gastric cancer datasets. The x-axis was generated according to the list of significant pathways in the gastric cancer data clustered by IGSA clustering. The y-axis was generated according to the list of cases in the gastric cancer data clustered by IGSA clustering (samples whose pathway expression values were more similar to the average expression values of normal samples were closer to the origin of the coordinate). The dots represent the marks for pathways whose expression values in cancer samples were higher than the average level. The color of the dots from blue to green represents the potential progression (mild to severe) of the cancer.

Furthermore, we analyzed the clustering of gastric cancer datasets. Through double clustering for both samples and significant pathways, we observed expression changes of pathways in different gastric cancer samples. As shown in Fig 8, among the cancer samples whose significant pathway expression scores were similar to the average expression values of normal samples, pathways such as DNA replication and base excision repair (the basic pathways for cancer) showed significant expression. Furthermore, pathways that are more specific to certain tissues (e.g., salmonella infection) tended to show higher expression when the samples were associated with more severe disease (i.e., the pathway expression scores of samples were very different from the average expression values of normal samples).

To further validate the performance of IGSA enrichment analysis, we applied the method to three cancer-related datasets based on third-layer GO gene sets, including BP (Biological Process) gene sets and MF (Molecular Function) gene sets. Figure D in S1 File was the result of the IGSA method compared with other enrichment methods, including DAVID, GSEA, GSVA, ssGSEA and Z-score. S6–S8 Tables show the list of the significant differentially expressed GO gene sets found by the six methods (the method SPIA cannot be used for GO enrichment analysis) in three cancer-related datasets and the related supporting papers. S9 and S10 Tables show the comparison of the six methods from two aspects, the accuracy and the proportion of significant GO gene sets supported by papers. Figure E in S1 File shows the clustering of samples in hepatitis datasets based on GO gene sets. Figure F in S1 File shows the comparison of IGSA clustering based on SMIC, Euclidean distance, Pearson's correlation and Spearman's rank correlation in ovarian cancer data. Figure G in S1 File shows the classification comparison of IGSA (p value of 0.008), HCBGO (hierarchical clustering based on GO gene sets) (p value of 0.209) and HCBG (hierarchical clustering based on genes) in ovarian cancer datasets (TCGA batch 9). Figure H in S1 File shows the survival analysis of ovarian cancer datasets (TCGA batch 9 and batch 40). Furthermore, we analyzed the double clustering of gastric cancer datasets too (Figures I and J in S1 File). All of the results were consistent with the results based on the pathways.

## Discussion

Traditional methods (e.g., DAVID, GSEA, SPIA etc.) focus on the analysis of gene expression. The enrichment analysis used with these methods is based on changes in gene expression between cases and controls, with only one value used to represent the expression of a gene in all samples (e.g., the ratio of the mean value in cases to the mean value in controls), discarding the information from diverse individual gene expression. Particularly, in the analysis of personalized medicine, the gene sets (e.g., pathways) found using traditional methods contribute only slightly to the analysis. Other methods (such as ssGSEA, GSVA, etc.) calculate the gene set scores for each sample, although most of these methods lack the information of gene set (e.g., pathway) expression for various reasons (e.g., ranking of expression, etc.).

However, IGSA, as distinct from traditional methods whose enrichment strategies focus on the elimination of the impact of heterogeneity between samples at the gene expression level, takes advantage of the heterogeneity of gene expression in samples and focuses on specific and significant gene sets, as a complement of these traditional methods. In clustering, IGSA stresses the similarity between the samples in quality rather than in quantity, which averts the over-fitting caused by focusing too much on the quantity of similarity, which commonly occurs when using traditional methods. Moreover, IGSA uses an accumulating clustering strategy, adding disease samples into sets according to the extent of possible deterioration, which can reveal the severity of disease in different samples and correlate the related changes in pathways with the severity of disease.

As mentioned in the results, compared with other methods (DAVID, GSEA, SPIA, GSVA, ssGSEA and Z-score), the performance of IGSA was comparatively specific, sensitive and robust. In clustering, IGSA proved to be more effective than the traditional method (unsupervised hierarchical clustering based on significant gene expression). The reason for this result is that IGSA concentrates on the similarity of samples at the functional level rather than at the molecular level, which is more susceptible to noise and samples. In the classification, the classes divided by IGSA are not very well separated (class 3 is not markedly separated from the other classes), which may be caused by incomplete survival information, among other reasons (e.g., many other factors, including lncRNA, miRNA, etc. may also influence the survival time of patients). In addition, using IGSA, we identified a signature (13 papers supporting SUPs and 16 paper supporting SDPs found by IGSA) for ovarian cancer, and the survival times of the classes based on the signatures were significantly distinct.

IGSA also provides the expression of significant gene sets in each sample, which reveals a legible difference in different classes and is conducive to personalized medicine. It is also possible for IGSA clustering to indicate the potential progression of a disease due to the strategy used for clustering, which may be very useful for the analysis of disease progression at the functional level.

To assess IGSA universality, we added other 3 non-cancer datasets from GEO for the analysis which includes diabetes (Type II) GSE9006, myocardial infarction GSE48060, and rheumatoid arthritis GSE55235. Except myocardial infarction, the performance of IGSA on the other two datasets is better. The accuracy of detecting significant pathways were as high as 100% for diabetes and 92.9% for rheumatoid arthritis, while the accuracy of detecting significant pathways for myocardial infarction was 62.5% (S11 Table).The results showed that IGSA is not only suitable for cancer analysis and is also applicable to other complicated diseases.

There are also some flaws with IGSA; for instance, this approach has some difficulty in finding pathways in datasets with a relatively low proportion of control samples. According to the results of enrichment, the proportion of controls in the samples has an impact on pathway identification. IGSA effectively identified pathways in gastric cancer and pancreatic cancer datasets (the proportion is 22.62% and 30.77%), whereas in the lower proportion samples (such as the ovarian cancer dataset, in which the control proportion was only 17.78%), the performance of IGSA was decreased. In spite of this, IGSA performed well in finding SUPs compared with the other methods. It is also difficult for IGSA to analyze data with few samples because under such conditions, the strategy adopted by IGSA for enrichment will retrograde to the traditional method (for instance, one case and one control).

In addition, relying on gene expression data alone to analyze cancer-related data is not sufficient; instead, it is more effective to integrate IGSA with other tools (suitable for other types of data, such as miRNA, CNVs, lncRNA, etc.) to analyze cancer samples. The strategy used in IGSA is also suitable for the analysis of other data (e.g., miRNA expression profiles).

At present, analyses based on integrating multiple genomic datasets are very common. Du et al. [25] analyzed clinically relevant lncRNAs in human cancer through the integration of SCNA (somatic copy number alteration), lncRNA and clinical data. Moreover, Wu et al. [26] predicted disease-causing nonsynonymous single-nucleotide variants by integrating multiple genomic data, and Sanchez et al. [27] integrated the analysis of Chip-seq and RNA-seq data to unveil a lncRNA tumor suppressor signature. In the future, we plan to expand the capacity of IGSA analysis and devise a framework for IGSA to analyze samples through integrating multiple datasets (including CNVs, SVs, lncRNAs, miRNAs, etc.).

## Supporting Information

S1 File (PDF)

S1 Table The significant differentially expressed pathways in gastric cancer data. (ZIP)

S2 Table The significant differentially expressed pathways in pancreatic cancer data. (ZIP)

S3 Table The significant differentially expressed pathways in ovarian cancer data. (ZIP)

S4 Table The accuracy of SUPs and SDPs in three cancer data. (ZIP)

S5 Table The average accuracy of seven methods. (ZIP)

S6 Table The significant differentially expressed GO gene sets in gastric cancer data. (ZIP)

S7 Table The significant differentially expressed GO gene sets in pancreatic cancer data. (ZIP)

S8 Table The significant differentially expressed GO gene sets in ovarian cancer data. (ZIP)

S9 Table The accuracy of SUPs and SDPs based on BP_3(MF_3) in gastric cancer data. (ZIP)

S10 Table The average ratios of SGSs based on OTERM_BP_3(GOTERM_MF_3) found by different methods, and the average ratios of SGSs found by different methods. (ZIP)

S11 Table The accuray of detecting significant pathways in three on-cancer dataset. (ZIP)
