# Supplementary material for: IGSA: Individual Gene Sets Analysis, including Enrichment and Clustering
Source: PLoS One. 2016 Oct 20;11(10):e0164542. doi: 10.1371/journal.pone.0164542 (PMC5072653; doi:10.1371/journal.pone.0164542)
Supplement: S1 File — (PDF) [file pone.0164542.s001.pdf]

IGSA: Individual Gene Sets Analysis, including enrichment and clustering

Lingxiang Wu[1¶], Xiujie Chen [1¶*], Denan Zhang[1¶], Wubing Zhang[1¶], Lei Liu[1], Hongzhe Ma[1], Jingbo Yang[1], Hongbo Xie[1], Bo Liu[1], Qing Jin [1]

[1]College of Bioinformatics Science and Technology, Harbin Medical University, Harbin, Heilongjiang, China

* Corresponding author

E-mail: chenxiujie@ems.hrbmu.edu.cn

¶ These authors contributed equally to this work.

Table of Contents

Part I The results of IGSA based on pathways

Figure A ………………………………………………………………….3

Figure B ………………………………………………………………….4

Figure C ………………………………………………………………….5

Part II The results of IGSA based on GO(Gene Ontology) gene sets

Figure D …………….…………………………………………………….6

Figure E ………………………….……………………………………….6

Figure F ……………………..…………………………………………….7

Figure G ……..………………..………………………………………….8

Figure H ………………………..………………………………………….8

Figure I ………………………...………………………………………….9

Figure J ……………………………………………………………………10

Supplements Information

Part I The results of IGSA based on pathways

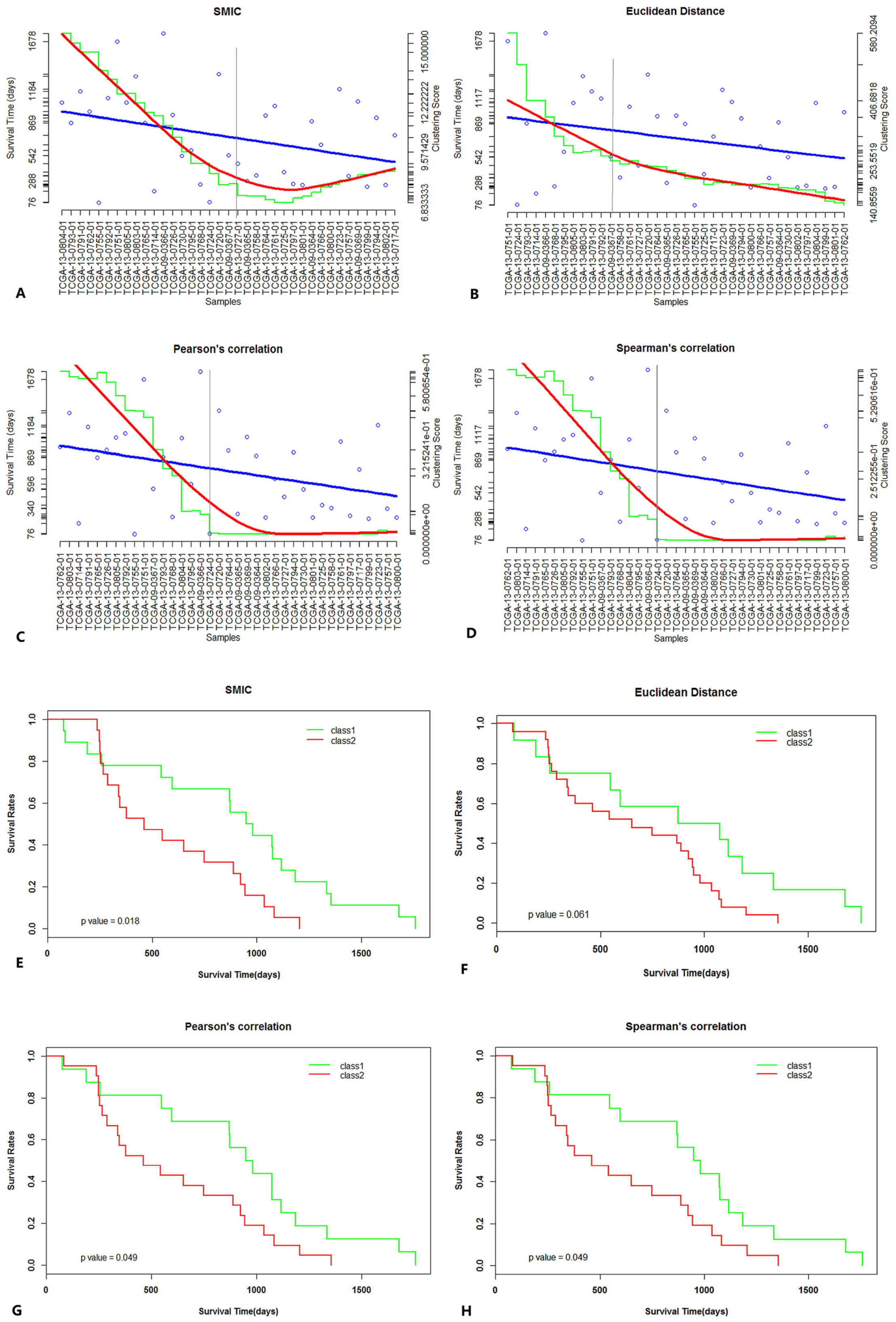

Figure A | The comparison of IGSA clustering with different similarity measurement. The blue points represent the survival time of the samples. The blue lines were generated by linear fitting the blue points. The red curves were loess curves obtained by fitting the similarity scores.

Supplements Information

The gray vertical lines are used to distinguish the samples according to the flex points in the red curve. The first four figures (A, B, C, D) represented the IGSA clustering based on SMIC, Euclidean distance, Pearson's correlation and Spearman's rank correlation, respectively, which were applied in the ovarian cancer data set (batch 9) based on pathways. The last four figures (E, F, G, H) represented the survival analysis studies on these classes that obtained by the IGSA clustering based on SMIC, Euclidean distance, Pearson's correlation and Spearman's rank correlation, respectively. The survival time in all the methods (A, B, C, D) tended to decrease. However, compared with Euclidean distance (F) (p value of 0.061), Pearson's correlation (G) (p value of 0.049) and Spearman's rank correlation (H) (p value of 0.049), the SMIC (E) (p value of 0.018) was more remarkable, although the clustering based on Pearson's correlation and Spearman's rank correlation can cluster the disease samples significantly too.

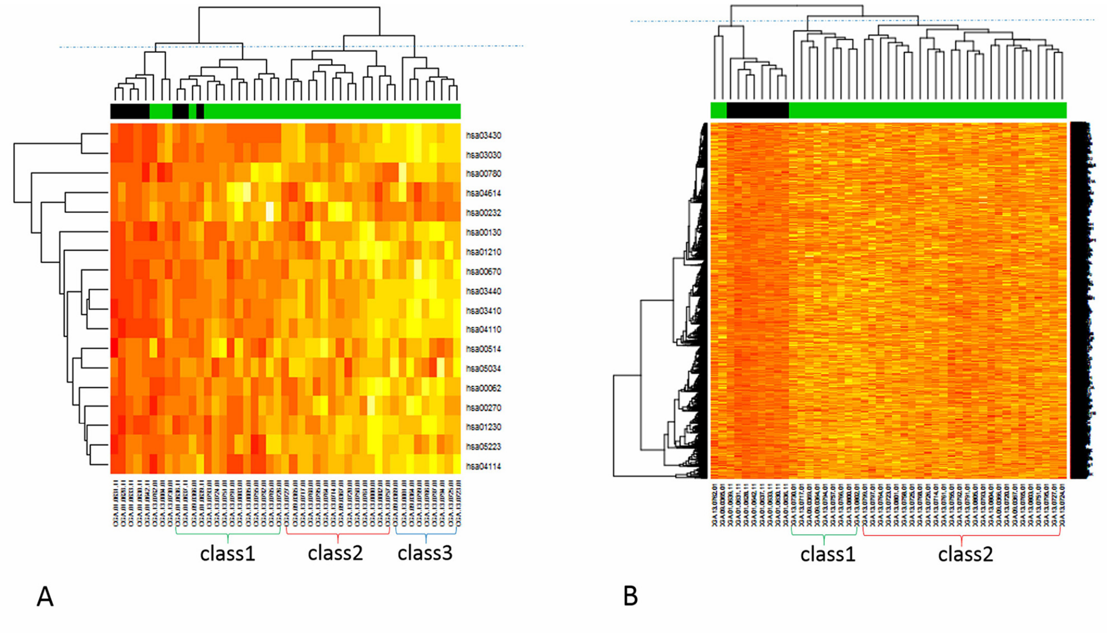

Figure B | The clustering of HCBP (hierachical clustering based on pathways) and HCBG (hierachical clustering based on genes) in ovarian cancer data. A represents hierachical clustering based on significant pathways (IGSA, FDR<0.25). B represents hierachical clustering based on significant genes (SAM, FDR<0.1). The dashed lines are the threshold for distinguishing the controls from the cases. The cancer samples are divided into different classes (three classes in A, two classes in B) according to threshold.

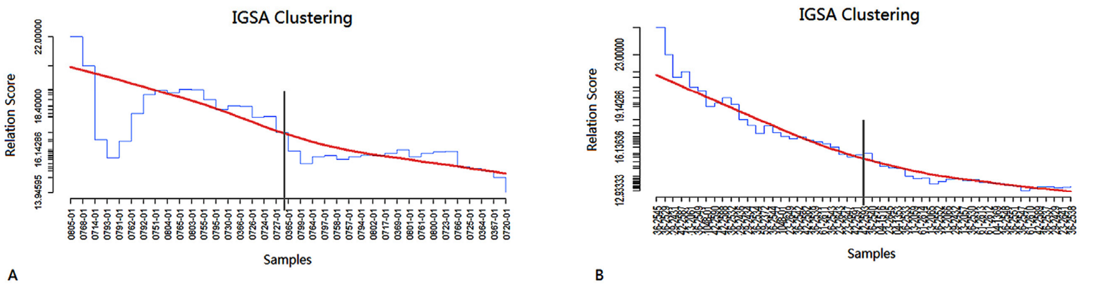

Figure C | The IGSA clustering of disease samples in ovarian cancer data (batch9 and batch 40). The blue curves show the average similarity scores of clustering samples. The red curves

were loess curves obtained by fitting the similarity scores. The gray vertical lines are used to divide the samples according to the flex points in the red curve. A represents the IGSA clustering applied in ovarian cancer data (batch 9). B represents the IGSA clustering applied in ovarian cancer data (batch 40). The disease samples in the two batch data were both divided into two classes, and according to the survival analysis, the difference between the two classes was significant (Figure 6 in article).The p value were 0.0778 and 0.0364 in batch 9 data and batch 40, respectively.

Part II The results of IGSA based on GO(Gene Ontology) gene sets

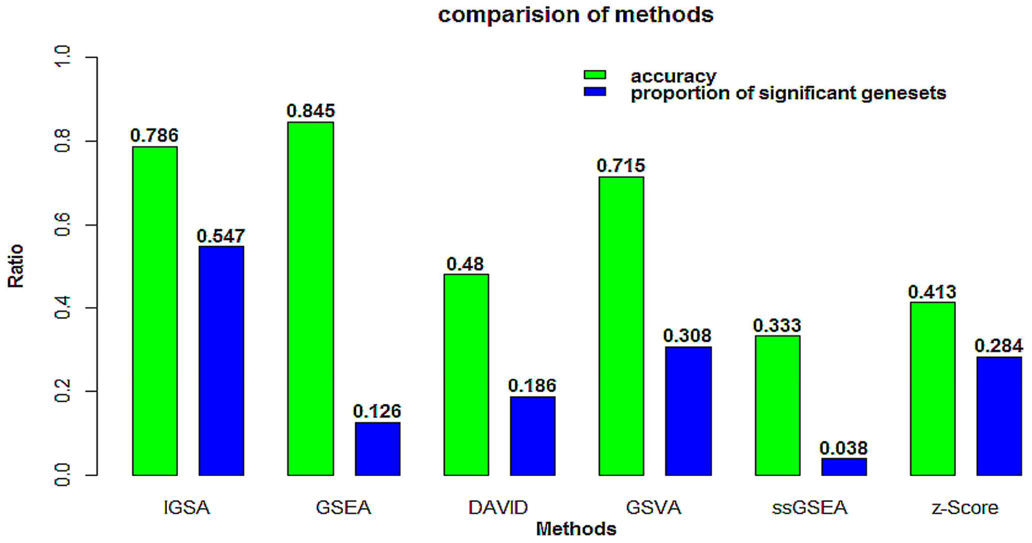

Figure D | The comparison of the accuracy of six enrichment analysis methods based on GO gene sets (the method SPIA cannot be used for GO enrichment analysis). The green columns represent the average accuracy in three cancer-related datasets of the six methods. The blue columns represent the proportion of significant GO gene sets supported by papers found in three cancer-related datasets. IGSA, compared with the other methods, can identify robust and sensitive significant GO gene sets for different cancer types. Although the average accuracy of DAVID was a bit higher than that of IGSA, the proportion of significant GO gene sets supported by papers found in three cancer-related datasets was very low, that meant DAVID found only a subset of significant GO gene sets.

Supplements Information

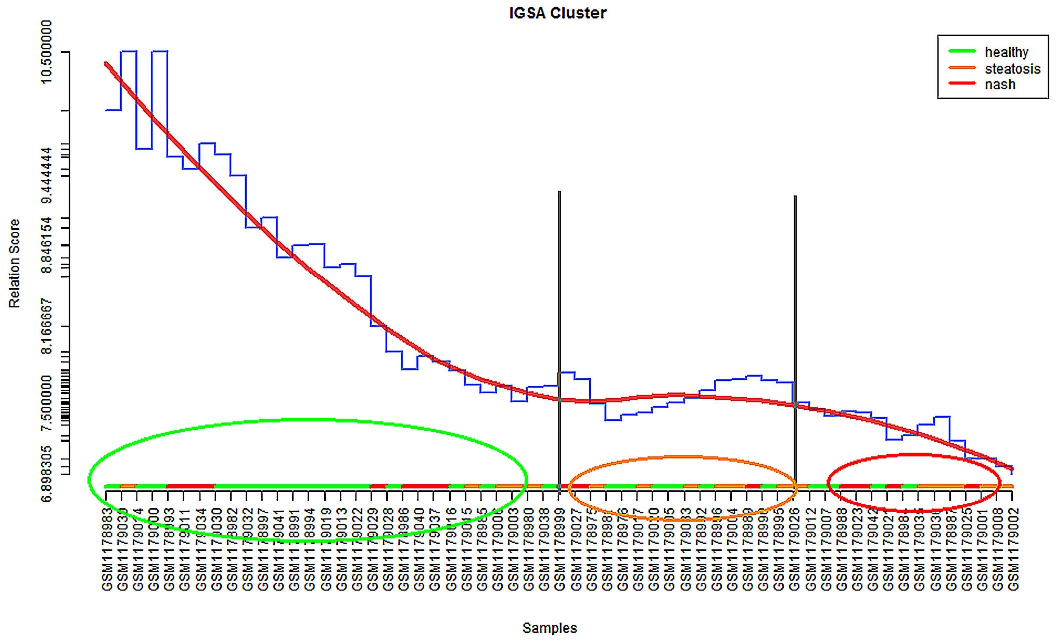

Figure E | The IGSA clustering of disease samples in hepatitis datasets based on GO gene sets (including BP gene sets and MF gene sets). The blue curves show the average similarity scores of clustering samples. The red curves were loess curves obtained by fitting the similarity scores. The gray vertical lines are used to divide the samples according to the flex points in the red curve. Most serious disease samples (nash) tended to be clustered on the right (in class 3). In class 2, Samples of less severe disease (steatosis) showed a tendency to cluster in the middle. In class 1, most of healthy obese samples tended to be clustered on the left. To some extent, the clustering may reveal the severity of samples in hepatitis datasets.

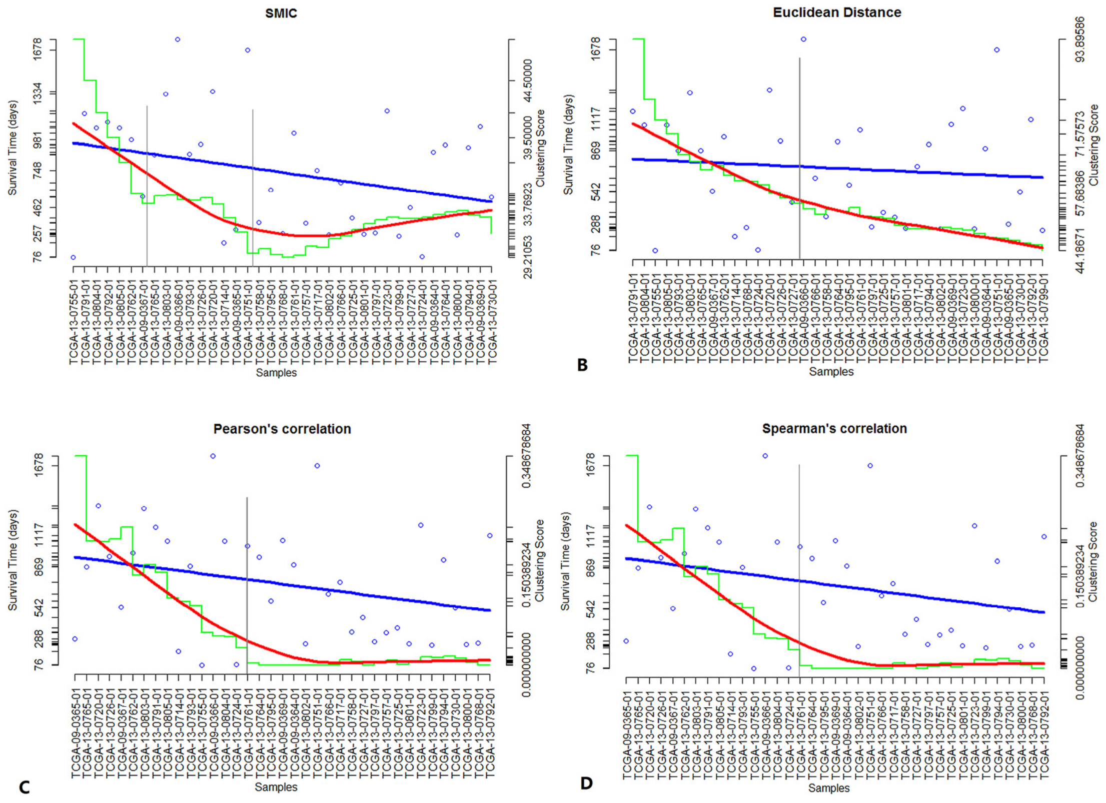

Supplements Information

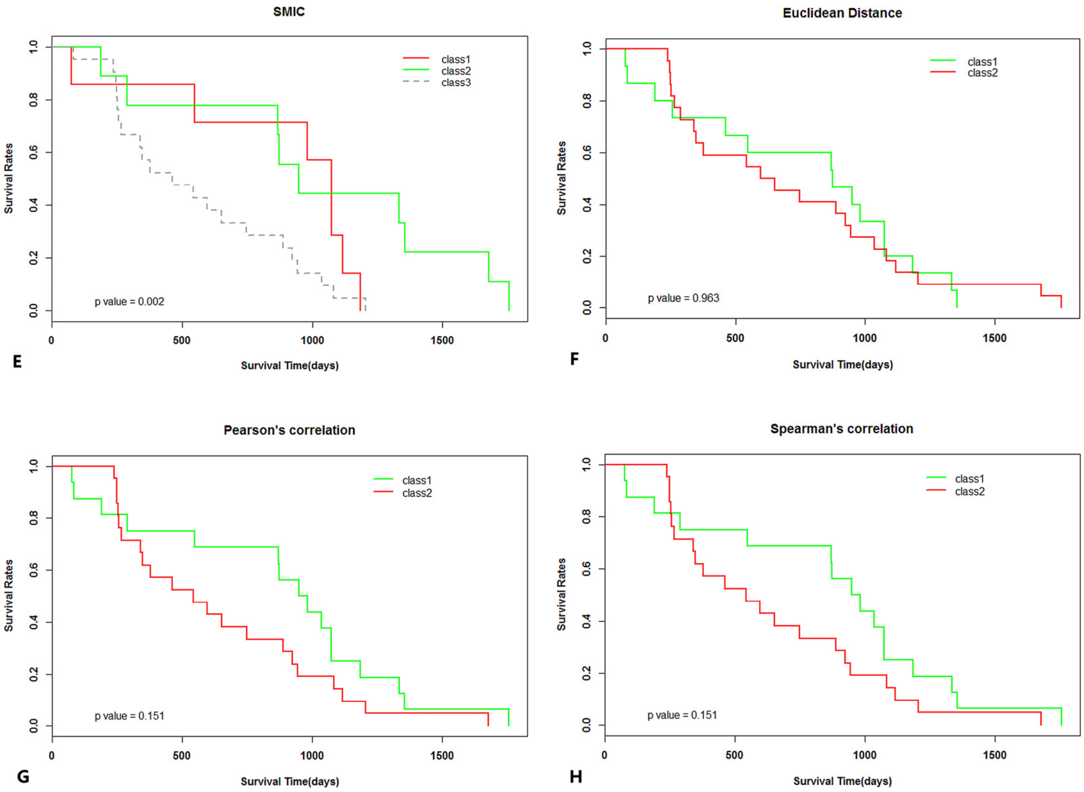

Figure F | The comparison of IGSA clustering with different similarity measurement. The blue points represent the survival time of the samples. The blue lines were generated by linear fitting the blue points. The red curves were loess curves obtained by fitting the similarity scores. The gray vertical lines are used to distinguish the samples according to the flex points in the red curve. The first four figures (A, B, C, D) represented the IGSA clustering based on SMIC, Euclidean distance, Pearson's correlation and Spearman's rank correlation, respectively, which were applied in the ovarian cancer data set (batch 9) based on GO gene sets (including BP and MF). The last four figures (E, F, G, H) represented the survival analysis studies on these classes that obtained by the IGSA clustering based on SMIC, Euclidean distance, Pearson's correlation and Spearman's rank correlation, respectively. The survival time in all the methods (A, B, C, D) tended to decrease. However, according to the results of survival analysis, the clustering based on SMIC (E) (p value of 0.002) divided the disease sample into three different classes significantly, while the clustering based on Euclidean distance (F) (p value of 0.963), Pearson's correlation (G) (p value of 0.151) and Spearman's rank correlation (H) (p value of 0.151) didn’t cluster the disease sample well.

Supplements Information

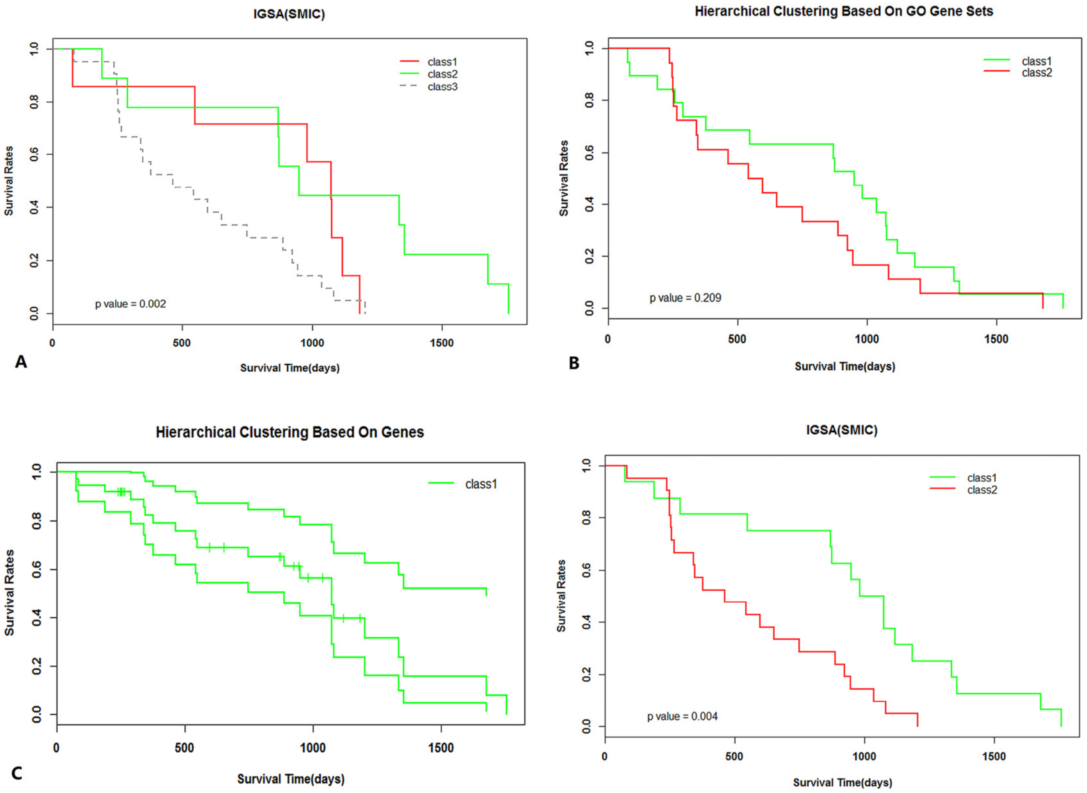

Figure G | The classification comparison of IGSA, HCBGO(hierarchical clustering based on GO gene sets) and HCBG (hierarchical clustering based on genes) in ovarian cancer datasets (TCGA batch 9). A shows the survival time curves of three classes obtained by IGSA (p value of 0.002). B shows the survival analysis of two classes obtained by HCBGO (p value of 0.209). C shows the survival time curves of one classes obtained by HCBG (hierarchical clustering based on genes can't classify the samples into different classes). D shows the survival time curves of two classes (class 3 and class 1, 2) obtained by IGSA (p value of 0.004). The p values in both A and D are significant compared with HCBP and HCBG.

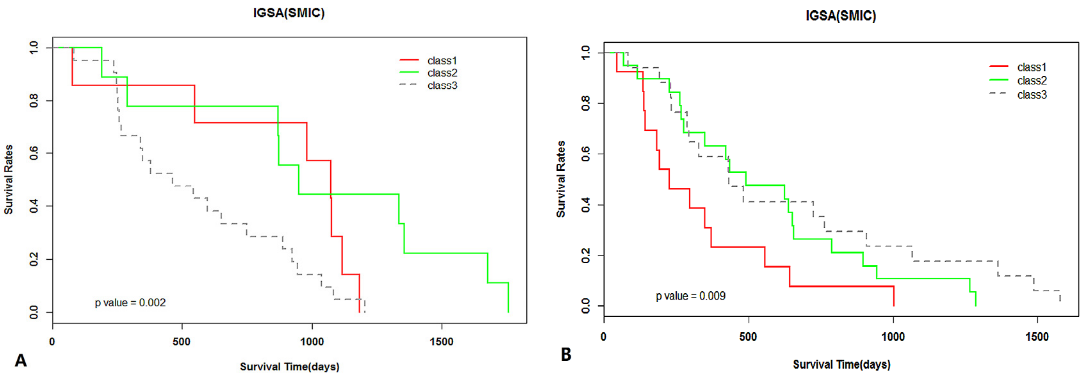

Figure H | The survival analysis of ovarian cancer datasets (TCGA batch 9 and batch 40) based on GO gene sets. A shows the survival time curves of three classes obtained by IGSA applied in the batch 9 ovarian cancer datasets (p value of 0.002). B shows the survival time curves of three classes obtained by IGSA applied in the batch 40 ovarian cancer datasets based on the same significant GO gene sets (p value of 0.009). Both the ovarian cancer datasets (batch9 and batch 40) can be classified into three classes significantly.

Supplements Information

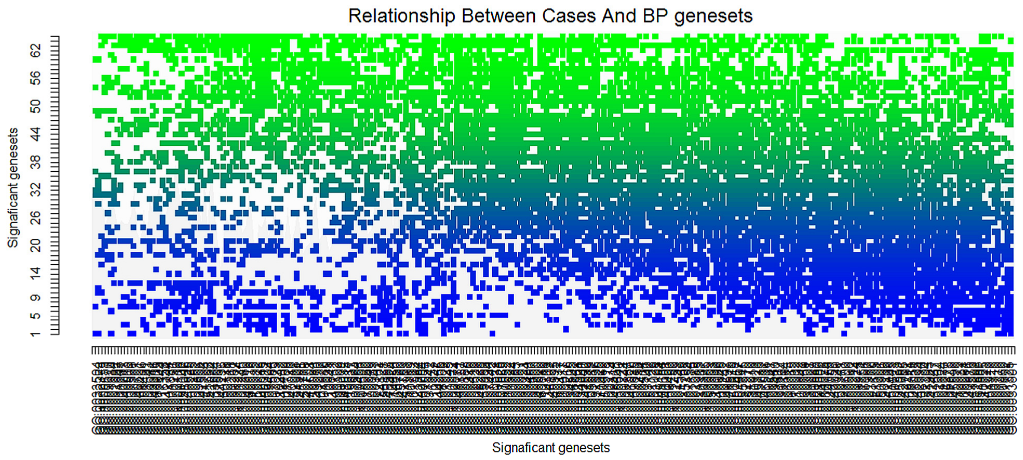

Figure I | The double clustering of samples and significant BP gene sets in gastric cancer datasets. The x-axis was generated according to the list of significant BP gene sets in the gastric cancer data clustered by IGSA clustering (samples whose BP gene sets expression values were more similar to the average expression values of normal samples were closer to the origin of the coordinate). The y-axis was generated according to the list of cases in the gastric cancer data clustered by IGSA clustering. The dots represent the marks for BP gene sets whose expression values in cancer samples were higher than the average level. The color of the dots from blue to green represents the potential progression (mild to severe) of the cancer.

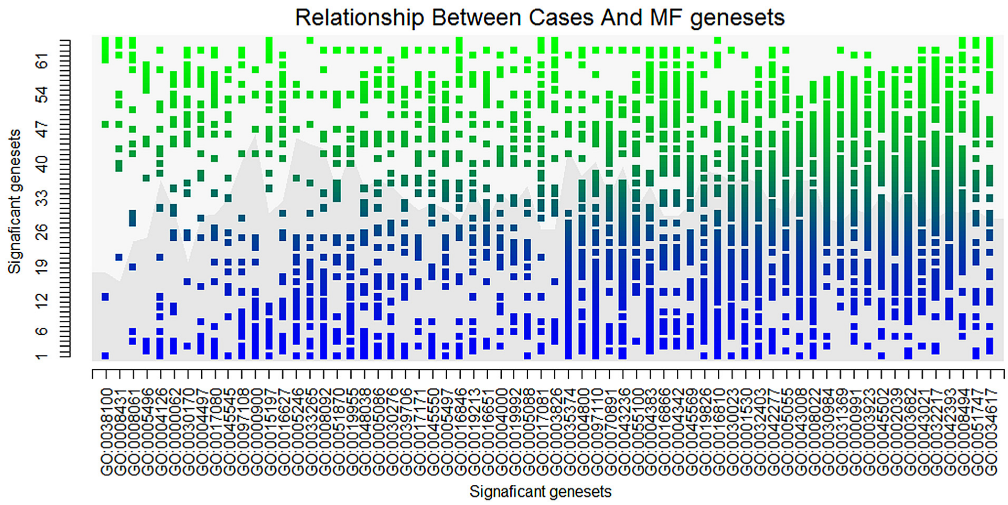

Figure J | The double clustering of samples and significant MF gene sets in gastric cancer datasets. The x-axis was generated according to the list of significant MF gene sets in the gastric cancer data clustered by IGSA clustering (samples whose MF gene sets expression values were more similar to the average expression values of normal samples were closer to the origin of the coordinate). The y-axis was generated according to the list of cases in the gastric cancer data clustered by IGSA clustering. The dots represent the marks for MF gene sets whose expression values in cancer samples were higher than the average level. The color of the dots from blue to

green represents the potential progression (mild to severe) of the cancer.
